# Factors Affecting Organelle Genome Stability in *Physcomitrella patens*

**DOI:** 10.3390/plants9020145

**Published:** 2020-01-23

**Authors:** Masaki Odahara

**Affiliations:** Biomacromolecules Research Team, RIKEN Center for Sustainable Resource Science, 2-1 Hirosawa, Wako-shi, Saitama 351-0198, Japan; masaki.odahara@riken.jp; Tel.: +81-48-462-1111

**Keywords:** chloroplast, mitochondrion, genome stability, homologous recombination repair, repeated sequence, *Physcomitrella patens*

## Abstract

Organelle genomes are essential for plants; however, the mechanisms underlying the maintenance of organelle genomes are incompletely understood. Using the basal land plant *Physcomitrella patens* as a model, nuclear-encoded homologs of bacterial-type homologous recombination repair (HRR) factors have been shown to play an important role in the maintenance of organelle genome stability by suppressing recombination between short dispersed repeats. In this review, I summarize the factors and pathways involved in the maintenance of genome stability, as well as the repeats that cause genomic instability in organelles in *P. patens*, and compare them with findings in other plant species. I also discuss the relationship between HRR factors and organelle genome structure from the evolutionary standpoint.

## 1. Introduction

*Physcomitrella patens* is a moss (bryophyte) that has been used as a model species for studying cell growth and differentiation [1]. Additionally, *P. patens* is recognized as a model for land plants because it is located at the base of the land plant lineage [2]. The life cycle of *P. patens* is simple and mostly haploid. Germinated spores of *P. patens* produce filamentous protonemal cells comprising chloronemal and caulonemal cells, which subsequently produce gametophores with leafy shoots. Sporophyte, the only diploid phase in the life cycle of *P. patens*, is developed from zygotes, archegonia, and antheridia, which are formed at the top of gametophores. Nuclear DNA of *P. patens* shows exceptionally high activity of homologous recombination, which enables its use for gene targeting in combination with polyethylene glycol-mediated protoplast transformation [3]. This feature, together with its haploid vegetative growth phase and recent advances in nuclear genome analysis, has accelerated reverse genetic analyses in *P. patens* [2,4].

Each *P. patens* cell harbors ≈50 large spindle-shaped chloroplasts and many rod- or sphere-shaped mitochondria. Chloroplast and mitochondria in *P. patens*, as in other plant species and algae, possess their own DNA, which associates with proteins to form nucleoids. The mitochondrial DNA (mtDNA) of *P. patens* is 105 kb in size and harbors genes encoding transfer RNAs (tRNAs), ribosomal RNAs (rRNAs), and proteins that regulate gene expression and oxidative phosphorylation [5]. The mapped mitochondrial genomes of angiosperms are larger than that of *P. patens*; however, they are shown to form complicated structures including linear, branched, and circular structures [6]. Moreover, homologous recombination between repeats longer than 1 kb, which are frequently observed in angiosperm mtDNA, makes them a more complicated structure. By contrast, *P. patens* mtDNA forms a single circular structure because of the absence of repeats longer than 80 bp [5,7,8,9]. The chloroplast DNA (cpDNA) of *P. patens* is 123 kb in size and contains genes encoding tRNAs, rRNAs, and proteins including subunits of RNA polymerase- and photosynthesis-related proteins [10]. The cpDNA of *P. patens* exhibits a typical circular structure with large single-copy (LSC) and small single-copy (SSC) regions separated by a pair of large inverted repeat (IR) regions [10]. Except for the large IR regions (9.6 kb each), the longest dispersed repeat in *P. patens* cpDNA is 63 bp in size, with a 3 bp mismatch [11]. Notably, neither mtDNA nor cpDNA encode proteins that are involved in DNA replication, recombination, and repair; instead, proteins involved in these processes are encoded by nuclear DNA, similar to a large number of proteins that function in chloroplasts and mitochondria.

## 2. Plant Homologs of Bacterial Proteins and Their Localization

Because chloroplasts and mitochondria are derived from bacteria, internal contents of these organelles resemble prokaryotes. Although orthologs of bacterial proteins function in chloroplasts and mitochondria, most of the chloroplast and mitochondrial proteins are encoded by nuclear DNA because of gene transfer during evolution. In bacteria, homologous recombination repair (HRR) proteins repair DNA double-strand breaks and collapsed or stalled replication forks. Homologs of bacterial HRR factors are also found in the nuclear genome of *P. patens* and that of other plant species. The N-terminus of HRR factors contain signal peptides that target these proteins to chloroplasts and/or mitochondria. Interestingly, such bacterial-type HRR factors have not been found in animal or yeast nuclear genomes [8,12,13,14], implying the existence of plant-specific mechanisms underlying organelle DNA maintenance by HRR. Table 1 summarizes plant homologs of bacterial HRR factors and MutS homolog 1 (MSH1; involved in organelle genome stabilization) in *P. patens* and other plant species, including *Chlamydomonas reinhardtii* and *Arabidopsis thaliana*, which are representative models of green algae and angiosperms, respectively. Nuclear genomes of *P. patens* and other plant species encode several homologs of bacterial HRR factors, although some homologs have not been identified in the genomes of *P. patens* and other plant species, on the basis of sequence similarity.

RecA is a key factor in HRR, as it binds to single-stranded DNA (ssDNA) and identifies homologous sequences to perform strand exchange between them [27]. Nuclear DNA of *P. patens* encodes two types of RecA homologs, RECA1 and RECA2, which show moderate sequence similarity. Phylogenetic analysis shows that these two RECA proteins cluster with either cyanobacterial RecA or proteobacterial RecA in separate clades, suggesting that these proteins have different origins, that is, RECA1 from α-proteobacteria, and RECA2 from cyanobacteria [13]. Products of *RECA1* and *RECA2* genes expressed from the nuclear DNA are predominantly localized to mitochondria and chloroplasts, respectively, thus reflecting their predicted origins [13,18]. When full-length RECA1 and RECA2 proteins are transiently produced in protoplasts, they form granular structures that associate with organelle nucleoids [8,18], indicating that these proteins constantly associate with and/or act on nucleoids. Consistent with this hypothesis, chloroplast RecA is shown to associate with the chloroplast nucleoid by nucleoids enriched proteome in maize [28]. Interestingly, although HRR factors are encoded by a single conserved gene in plants, the copy number of *RECA* varies among plant species. Although *A. thaliana* and other flowering plants harbor multiple copies of the *RECA* gene, and the encoded proteins localize to chloroplasts and/or mitochondria, algae, including *C. reinhardtii*, harbor a single *RECA* gene copy, and the encoded RecA homolog localizes to chloroplasts [12] (Table 1).

RecG, a DNA helicase/translocase, functions in the rescue of branched DNA structures including stalled replication forks [29]. The nuclear genome of *P. patens* harbors a single copy of the *RECG* gene [14]. Phylogenetic analysis shows that plant RecG homologs, including *P. patens* RECG, are closely related to cyanobacterial RecG, suggesting that these proteins originated from cyanobacteria [23]. The RECG protein of *P. patens* harbors an ambiguous N-terminal signal peptide but localizes to both chloroplasts and mitochondria, similar to the *A. thaliana* RecG homolog, RECG1 [14,23]. Moreover, full-length *P. patens* RECG protein localizes to nucleoids of both organelles [14].

Unlike RecA and RecG, RecX does not act directly on DNA but participates in HRR by directly regulating RecA activity [30]. Although RecX is absent from several bacterial classes including α-proteobacteria and cyanobacteria [31], it is encoded by single copy genes present in the nuclear genomes of diverse plants ranging from green algae to angiosperms [8]. Because of difficulty in analyzing the evolutional origin of plant RecX homologs, it is unclear whether α-proteobacteria and cyanobacteria lost their RecX or plants acquired RecX via horizontal gene transfer. In protoplasts, a fluorescent protein-tagged RecX homolog of *P. patens*, RECX, localizes to mitochondrial and chloroplast nucleoids, thereby co-localizing with RECA1 and RECA2, respectively [8].

MSH is a eukaryotic homolog of bacterial MutS. Among several types of MSH proteins, MSH1 is the only protein that localizes to organelles [32,33]. MSH1 was originally identified in *A. thaliana* as a chloroplast mutator (CHM) protein because of the variegated phenotype of the mutant [34,35]. MSH1 is distinct from other MSH proteins and MutS because of the presence of the GIY-YIG endonuclease domain at its C-terminal end [21]. The nuclear genome of *P. patens* harbors two *MSH1* genes, *MSH1A* and *MSH1B*, although nuclear genomes of other plants carry only one *MSH1* gene copy. Because MSH1A lacks the C-terminal endonuclease domain, *P. patens MSH1* genes are thought to be derived by gene duplication or the loss of C-termini endonuclease domains after the duplication event [25]. Both *P. patens* MSH1 proteins (MSH1A and MSH1B) localize to organelle nucleoids by forming granular structures [25], similar to the MSH1 localization pattern in *A. thaliana* [26].

## 3. Maintenance of Mitochondrial Genome Stability by HRR and MSH1

### 3.1. RECA

*P. patens* mitochondrial *RECA1* knockout (KO) mutants generated by targeted gene disruption show severe defects in protonema cells, with less-developed gametophores and defective mitochondria characterized by an enlarged shape, disorganized cristae, and lower matrix electron density [7], indicating that *RECA1* is essential for normal growth. The mitochondrial genome of *P. patens RECA1* KO mutant is destabilized by the accumulation of products derived from aberrant recombination between short repeats dispersed throughout the mtDNA [7]. Most of the 24 pairs of repeats (≥30 bp) identified in *P. patens* mtDNA are involved in recombination in *RECA1* KO plants [8], occasionally leading to the generation of subgenomes [7]. Interestingly, because most of the repeats are located in introns of genes in the direct orientation, recombination between them leads to the loss of genes and generation of subgenomes, which may be subsequently lost, as these are not replicated. Thus, copy number variation of loci resulting from the loss of subgenomes is associated with instability of mtDNA in the *RECA1* KO mutant [14]. Collectively, these findings show the role of RECA1 in maintaining mtDNA stability by suppressing aberrant recombination between short dispersed repeats (SDRs) in *P. patens*. Additionally, defects in the recovery of mtDNA damaged by methyl methanesulfonate (MMS) in *RECA1* KO plants suggest the involvement of RECA1 in the repair of exogenously damaged mtDNA [13].

In *A. thaliana*, two RecA homologs, RECA2 and RECA3, localize to mitochondria (Table 1). In comparison with RECA2, RECA3 is more diverged from other RECAs and has truncated C-terminus, which is considered unusual because the C-terminus of RecA is important for its function [21,36]. Consistent with the gene structure, *A. thaliana RECA2* mutants are seedling-lethal, thus indicating the importance of RECA2 for normal plant growth; by contrast, *RECA3* mutants are almost indistinguishable from the wild type [21]. Both *RECA2* and *RECA3* mutants accumulate products derived from recombination between intermediate-sized (100–300 bp) repeats in mtDNA, and the number of repeats involving recombination in *RECA2* mutants exceed that of *RECA3* mutants [36]. Although recombination between shorter repeats (<100 bp) has not been tested in *A. thaliana RECA2* and *RECA3* mutants, the aforementioned findings suggest a fundamental role of plant mitochondrial RecA homologs in maintaining mitochondrial genome stability by suppressing aberrant recombination between short repeats.

### 3.2. RECG

KO mutation of *P. patens RECG* gene leads to growth and morphological defects that are similar to but milder than those caused by the KO mutation of *RECA1* in plants [14]. The *RECG* KO mutant plants exhibit abnormal mitochondria, with disorganized cristae and lower matrix density. Moreover, mtDNA of the *RECG* KO mutant is destabilized by SDR-mediated recombination, similar to the mtDNA of the *RECA1* KO mutant, and the length of repeats involved in recombination is also similar between *RECA1* and *RECG* KO mutants [14]. However, these repeats exhibit some differences between *RECA1* and *RECG* KO mutants; for example, at the mitochondrial *atp9* locus, recombination between *ccmF* and *atp9* mediated by 47 bp repeats leads to product accumulation in mitochondria of the *RECG* KO mutant, whereas recombination between *nad2* and *atp9* mediated by 60 bp repeats, which is a hallmark of recombination induced by the *RECA1* KO mutation [7], does not lead to product accumulation in mitochondria of the *RECG* KO mutant [14]. Furthermore, increase in copy numbers of all tested loci in the *RECG* KO mutant differed from that in the *RECA1* KO mutant. These differences suggest that RECG of *P. patens* plays a somewhat different role from that of RECA1 in the maintenance of mtDNA stability. Because the amount of mitochondrial recombination products often show a direct correlation with the heterogeneous *RECG* KO growth defects, recombination between mitochondrial SDRs is considered as the cause of all morphological phenotypes [14]. Because of mtDNA rearrangements induced by the KO mutation of *RECG*, the level of mitochondrial transcripts is decreased by recombination between repeats located in introns of mitochondrial genes [14]. Although *A. thaliana RECG1* mutants are morphologically indistinguishable from wild-type plants under normal growth conditions, they show mtDNA instability because of aberrant recombination between intermediate-sized repeats (100–500 bp in length) [23]. Thus, RECG1 participates in the suppression of recombination between intermediate-sized repeats, and the loss of *RECG1* leading to the accumulation of recombination products. Although recombination between shorter repeats has not been analyzed in *A. thaliana RECG1* mutants, recombination surveillance indicates that RecG homolog is involved in the suppression of aberrant recombination between short and/or imperfect repeats in plant mitochondria.

### 3.3. RECX

KO mutation of *P. patens RECX*, which leads to no significant morphological phenotypes, results in a minor but reliable increase in products derived from recombination between several pairs of mitochondrial SDRs [8], suggesting the involvement of RECX in the maintenance of mtDNA stability. Overexpression (OEX) of *P. patens RECX* in plants leads to mtDNA instability because of the induction of recombination between many pairs of SDRs, sometimes with a comparable level with mtDNA instability in the *RECA1* KO mutant [8]. Taking into account the protein–protein interaction between *P. patens* RECX and RECA1, as revealed by yeast two-hybrid assays, RECX is believed to modulate the function of RECA1 by directly binding to RECA1 to maintain mtDNA stability, rather than inducing mtDNA instability in wild type. The involvement of *RECX* in the maintenance of mtDNA stability is also supported by the positive correlation between the expression of *RECX* and other mtDNA stabilizing genes, including *RECA1* and *RECG*, in several tissues of *P. patens* [8]. Interestingly, the expression of *RECX*, *RECA1*, *RECG*, and *MSH1B* is highly increased in *P. patens* spores, thus indicating their roles in mtDNA maintenance during transmission to progenies.

### 3.4. MSH1

Because *P. patens* unusually possesses two *MSH1* genes, single and double KO mutants of *MSH1* genes were generated. Although the single and double *MSH1* mutants showed no significant phenotypes compared with the wild type, comparison among the mutants show an involvement of *MSH1B* in the maintenance of mtDNA [25]. In the single *MSH1B* KO mutant and *MSH1A* and *MSH1B* double KO mutants, mtDNA is similarly destabilized by the induction of recombination between mitochondrial repeats (21–69 bp in length) that overlap with those in *P. patens RECA1* or *RECG* KO mitochondria. On the other hand, the accumulation of products derived from recombination between *nad2* and *atp9*, rather than that of products derived from recombination between *ccmF* and *atp9*, hallmarks of the mitochondrial *atp9* locus in *RECA1* KO and *RECG* KO mutants, respectively, in the *MSH1B* mutant suggest a similar mechanism of mtDNA stabilization between MSH1B and RECA1, whereas the *MSH1 RECA1* double KO mutant is likely lethal [25]. Genetic interaction between *P. patens MSH1B* and *RECG* loci, as shown by epistatic analysis of the suppression of recombination, suggests that MSH1B and RECA1 act in distinct pathways that converge at a node in mitochondria [25]. The importance of the GIY-YIG endonuclease domain of MSH1 for the suppression of recombination is indicated by its deletion mutants; on the other hand, no significant phenotypes are observed in the *MSH1A* KO mutant*,* which lacks the endonuclease domain [25]. The instability of mtDNA in *A. thaliana MSH1* mutants is well characterized; in these mutants, recombination is observed between 50–556 bp repeats, and the length of these repeats overlaps with that of repeats responsible for mtDNA instability in the *P. patens MSH1B* KO mutant [21,32,37]. Moreover, the difference in mtDNA rearrangements between *A. thaliana MSH1* mutants and *RECA3* mutants, as well as the highly pronounced phenotypes of the *MSH1 RECA3* double KO mutants, suggest that these genes act in distinct but overlapping pathways [21]. Recent biochemical characterization of the GIY-YIG domain of *A. thaliana* MSH1 shows its binding to a branched DNA structure, proposing a mechanism for the suppression of recombination between repeats [38].

## 4. Maintenance of Chloroplast Genome Stability by HRR Proteins and MSH1

### 4.1. RECA

KO mutation of *P. patens RECA2* results in modest growth inhibition under glucose-deficient conditions and increased sensitivity to MMS or ultraviolet (UV) radiation, leading to DNA damage [11]. These phenotypes of the *RECA2* KO mutant are in contrast to those of the *RECA1* KO mutant of *P. patens*, which show severe growth defects under normal conditions. However, despite the slight effect of *RECA2* KO mutation on the morphology of *P. patens*, the cpDNA of the *RECA2* KO mutant is destabilized by the induction of recombination between SDRs (13–63 bp in length) [11]. This shows that RECA2 is involved in the maintenance of chloroplast genome stability by suppressing recombination between SDRs. Moreover, roles of RECA1 and RECA2 in mitochondria and chloroplasts suggest the common role of RecA homologs in maintaining organelle genome stability by suppressing aberrant recombination between SDRs. Because *P. patens* cpDNA has fewer relatively long (>35 bp) repeats, the lack of RecA homologs may lead to a slight effect on the stability of cpDNA compared with that of mtDNA. Impaired recovery of damaged cpDNA, but not that of nuclear DNA or mtDNA, in *P. patens RECA2* KO mutants suggests another role of RECA2 in the maintenance of cpDNA stability by promoting recovery from DNA damage [11]. In contrast to the modest phenotypes of *P. patens* lacking chloroplast RECA, the deficiency of chloroplast RECA (RECA1) in *A. thaliana* plants (Table 1) is lethal [21]. *A. thaliana* T-DNA insertion *RECA1* mutants in which the level of *RECA1* transcripts is decreased to 15% of that in the wild type suggest that RECA1 is involved in the maintenance of cpDNA integrity by maintaining the quantity and multimeric structure of cpDNA [39]. *A. thaliana* RECA1 also maintains cpDNA stability by preventing cpDNA rearrangements in plants carrying a mutation in *Whirly* genes, which encode a family of ssDNA-binding proteins that suppress cpDNA rearrangements [40,41]. Chloroplast RECA in *C. reinhardtii* (Table 1) is also involved in the maintenance of chloroplast genome stability by suppressing aberrant recombination between SDRs, and it regulates the dynamics of chloroplast nucleoid including segregation [42].

### 4.2. RECG

Because the morphological defects of *RECG* KO mutant plants are similar to those of *RECA1* KO mutant plants, the defects of *RECG* KO plants are mainly attributed to defects in mtDNA. However, KO mutation of *RECG* leads to abnormal chloroplasts that over-accumulate starch and possess less-developed thylakoids, implying defects in chloroplast function [14]. Indeed, cpDNA and mtDNA of the *RECG* KO mutant are destabilized by the induction of recombination between SDRs. The repeats involved in recombination are almost common between the cpDNA of *RECG* and *RECA2* KO mutants, although the accumulation of recombination products is higher in the *RECG* KO mutant than in the *RECA2* KO mutant [14]. These results suggest that RECG maintains chloroplast genome stability by suppressing recombination between a broad range of repeats in cpDNA. Both synergistic and suppressive relationships are observed between *RECG* and *RECA2*, with respect to the suppression of recombination between chloroplast repeats, depending on the type of repeats [25], suggesting a complex relationship between these genes. Thus, *RECG* and *RECA2* may act in distinct pathways or in the same pathway, depending on the repeats, to suppress recombination. *A. thaliana* RECG1 localizes to chloroplasts; however, evidence indicating the involvement of RECG1 in the maintenance of chloroplast genome stability is lacking [23].

### 4.3. RECX

Although RECX localizes to chloroplast nucleoids, significant phenotypes have not been observed in the chloroplasts of *P. patens RECX* KO mutants and OEX plants. These KO and OEX plants show a basal level of products derived from recombination between chloroplast SDRs, in contrast to *P. patens RECA2* KO plants, which accumulate these recombinant products to high levels [8]. However, yeast two-hybrid assays show protein–protein interaction between *P. patens* RECX and RECA2, which is stronger than that between RECX and RECA1 [8]. This implies that RECX may interact with RECA2 and modulate its activity to maintain chloroplast genome stability, and the effect of *RECX* KO mutation or OEX was not evident probably because of the moderate effect of RECA2 inhibition on cpDNA.

### 4.4. MSH1

**Figure 1:**
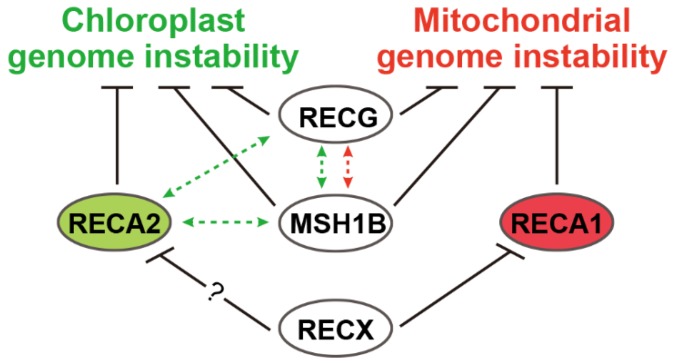
Factors affecting organelle genome stability in *P. patens*. Factors involving organelle genome stability are summarized with their relationship. Protein localization of the factors are shown by their colors: green (chloroplasts), red (mitochondria), and white (chloroplasts and mitochondria). Suppression and genetic relationship are shown by solid and dashed lines, respectively. RECX shows protein–protein interaction with RECA2, but its involvement in chloroplast genome stability remains unclear.

Similar to the instability of mitochondrial genome in the *MSH1* KO mutant, the *MSH1B* KO mutant shows chloroplast genome instability because of recombination between 28–63 bp SDRs in *P. patens* [25]. KO mutation of the *MSH1A* gene does not increase the abundance of recombination products in the wild-type or *MSH1B* KO mutant, indicating that *MSH1B* plays a predominant role in the suppression of recombination between SDRs in chloroplasts and mitochondria [25]. Interestingly, the level of recombination products in chloroplasts vary among the *P. patens MSH1B*, *RECA2*, and *RECG* KO mutant plants, depending on the type of repeats. Among these KO mutants, the level of products resulting from recombination between direct repeat-1 (DR-1) is the highest in *RECG* KO mutants, whereas the level of products resulting from recombination between inverted repeat-1 (IR-1) is the highest in *MSH1B* KO mutant plants [25]. This suggests a complicated regulation of recombination in chloroplasts. Similar complicated regulation is also observed in the genetic interaction between genes, as shown by synergistic relationships between *MSH1B* and *RECG* and between *MSH1B* and *RECA2*, although synergistic relationships have been observed for DR-1 but not for IR-1 [25]. Figure 1 summarizes all the factors affecting organelle stability and their relationship in *P. patens*. In *A. thaliana MSH1* mutants, cpDNA rearrangements at a locus containing a number of small repeats (<15 bp) indicate the involvement of MSH1 in maintaining chloroplast genome stability, although the details of these rearrangements remain unclear [26].

## 5. Organelle Genome Structure, Repeats, and HRR Proteins

Recent evidence in various plant species suggests the role of HRR factors in chloroplasts and mitochondria exclusively for the maintenance of genome stability by suppressing recombination between ectopic loci containing repeats, as summarized above. Because the phenomena of genome destabilization are common between mutants of organelle HRR factors, these factors likely function in a same suppression pathway. However, epistatic analyses of recombination suppression sometimes show that these factors act in distinct pathways [25]. Plant organelle HRR factors are thought to function in the repair of stalled or collapsed replication forks, which are prone to rearrangements in mutants [7]. Because such stalling and collapse of replication forks are caused by various types of DNA damage, the pathways of suppression in organelles may be regulated in a complicated manner. On the other hand, as shown in Table 1, not all HRR factors are conserved in plants, and some are absent in organelles of certain plant species; for example, mitochondrial RecA homologs are absent in some algae including *C. reinhardtii*, whereas copy numbers of mitochondrial RecA homologs are increased in various angiosperms including *A. thaliana* (Table 1) [12,13]. By contrast, chloroplast RecA copy numbers are conserved in plants (Table 1). Interestingly, the size and shape of mitochondrial genomes vary among plant species—*C. reinhardtii* possesses a 16 kb linear mitochondrial genome, whereas *A. thaliana* harbors a 368 kb multi-chromosome circular mitochondrial genome (Table 2). Moreover, the number of short repeats, which may lead to organelle genome instability because of the loss of HRR, corresponds to the size of the mitochondrial genome (Table 2). The presence/absence of RecA homologs may be correlated to the number and characteristics of repeats; RecA homologs are absent in algae because of the lack of significant repeats in mtDNA, whereas those in angiosperms are duplicated and functionally divergent to regulate recombination between increased and divergent repeats, or duplication of mitochondrial RecA homologs enabled increase of number of repeats in angiosperms. Recent advances in genome sequencing of various plant species provide an opportunity for exploring the relationship between HRR factors and organelle genome structure.

## Figures and Tables

**Table 1:** Summary of homologous recombination repair (HRR) factors and MutS homolog 1 (MSH1) in *Escherichia coli* and their plant homologs.

*E. coli*		*Physcomitrella patens*	*Arabidopsis thaliana*	*Chlamydomonas reinhardtii*
Protein	Function [15,16,17]	Protein/Localization		
RecFOR	Single-stranded DNA (ssDNA) binding	-	-	-
	RecA loading			
RecBCD	DNA Helicase/exonuclease	-	-	-
	RecA loading			
RecA	Homology search Strand exchange	RECA1/mt [13] RECA2/cp [18]	RECA1/cp [19]	REC1/cp [20]
			RECA2/cp, mt [21]	
			RECA3/mt [22]	
RecX	RecA regulation	RECX/cp, mt [8]	RECX	RECX
RecG	DNA Helicase/translocase	RECG/cp, mt [14]	RECG1/cp, mt [23]	-
RuvAB	Branch migration	-	-	-
RuvC	Holiday junction resolution	MOC1	MOC1/cp [24]	MOC1/cp [24]
MutS	Mismatch recognition	MSH1A/cp, mt [25]	MSH1/cp, mt [26]	MSH1
		MSH1B/cp, mt [25]		

**Table 2:** Genome size and number of repeats in organelle.

Organelle	Feature	*C. reinhardtii*	*P. patens*	*A. thaliana*
Chloroplast	Genome size (bp)	203,828 [43]	122,890 [10]	154,478 [44]
	Number of repeats	>5000	55	31
Mitochondrion	Genome size (bp)	15,758 [45]	105,340 [5]	367,808 [46]
	Number of repeats	3	136	507

Repeats identified as ≥20 bp of direct or inverted repeats without mismatch by using REPuter [47].
